# A phase II trial of docetaxel and erlotinib as first-line therapy for elderly patients with androgen-independent prostate cancer

**DOI:** 10.1186/1471-2407-7-142

**Published:** 2007-07-27

**Authors:** Mitchell Gross, Celestia Higano, Allan Pantuck, Olga Castellanos, Erica Green, Koo Nguyen, David B Agus

**Affiliations:** 1 Louis Warschaw Prostate Cancer Center, Cedars-Sinai Medical Center, Los Angeles, CA 90048, USA; 2 Department of Medicine, University of Washington, Seattle Cancer Care Alliance, Seattle, WA 98109, USA; 3 Department of Urology, David Geffen School of Medicine at UCLA, Los Angeles, CA, 90095, USA

## Abstract

**Background:**

Docetaxel is the standard first-line agent for the treatment of androgen-independent prostate cancer (AIPC). The combination of docetaxel with molecularly targeted therapies may offer the potential to increase the efficacy and decrease the toxicity of cytotoxic chemotherapy for prostate cancer. Previous studies demonstrate activation of the human epidermal growth factor receptor (EGFR) in prostate cancer. Erlotinib is a specific inhibitor of the tyrosine-kinase activity of EGFR. The goal of this study is to determine the anti-cancer activity docetaxel combined with erlotinib for the treatment of elderly subjects with AIPC.

**Methods:**

This is a multi-institutional Phase II study in patients with histologically confirmed adenocarcinoma of the prostate and age ≥ 65 years. Patients were requred to have progressive disease despite androgen-deprivation therapy as determined by: (1) measurable lesions on cross-sectional imaging; (2) metastatic disease by radionucleotide bone imaging; or (3) elevated prostate specific antigen (PSA). Treatment cycles consisted of docetaxel 60 mg/m^2 ^IV on day 1 and erlotinib 150 mg PO days 1–21. Patients with responding or stable disease after 9 cycles were eligible to continue on erlotinib alone as maintenance therapy.

**Results:**

Characteristics of 22 patients enrolled included: median age 73.5 years (range, 65–80); median Karnofsky Performance Status 90 (range 70–100); median hemoglobin 12.1 g/dl (range, 10.0–14.3); median PSA 218.3 ng/ml (range, 9–5754). A median of 6 treatment cycles were delivered per patient (range 1–17). No objective responses were observed in 8 patients with measurable lesions (0%, 95% CI 0–31%). Bone scan improvement and PSA decline was seen in 1 patient (5%, 95% CI 0.1–25%). Five of 22 patients experienced ≥ 50 % decline in PSA (23%, 95% CI 8–45%). Hematologic toxicity included grade 3 neutropenia in 9 patients and neutropenic fever in 2 patients. Common non-hematologic toxicities (≥ grade 3) included fatigue, anorexia, and diarrhea.

**Conclusion:**

Docetaxel/erlotinib can be delivered safely in elderly patients with AIPC. Anti-cancer disease activity appears generally comparable to docetaxel when used as monotherapy. Hematologic and non-hematologic toxicity may be increased over docetaxel monotherapy. Prospective randomized studies would be required to determine if the toxicity of docetaxel and erlotinib justifies its use in this setting.

## Background

Prostate cancer is a significant cause of cancer morbidity and mortality with a projected U.S. incidence of 230,000 cases diagnosed and 27,000 deaths in 2006 [1]. The progression to androgen-independent prostate cancer (AIPC) marks a stage of clinical acceleration to a lethal form of the disease. Mitoxantrone is commonly used for AIPC based on improvement in pain measures with no effect on overall survival [2,3]. Two large Phase III trials recently demonstrated a survival advantage for docetaxel over mitoxantrone and have led to the widespread use of docetaxel as first-line treatment for AIPC [4,5].

The human epidermal growth factor receptor (EGFR) tyrosine kinases are part of a network of pathways which are implicated in the development and progression of prostate cancer, in particular as part of the progression to androgen-independent growth [6-10]. Erlotinib is a selective inhibitor of the tyrosine kinase activity of the EGFR. Erlotinib was approved by the US Food and Drug Administration as a first-line therapy (in combination with gemcitabine) for pancreatic cancer and as second or third-line therapy for non-small cell lung cancer (NSCLC) based on improvement in overall survival [11,12].

The combination of pre-clinical and clinical data linking the HER-kinase axis to AIPC and early clinical data which demonstrated the safety of erlotinib in combination with docetaxel on a three-week schedule led us to perform this clinical study to examine the efficacy of erlotinib with docetaxel in AIPC [13].

A significant portion of patients treated with metastatic AIPC are elderly. The median patient age in the recent multi-center trials of first-line chemotherapy for AIPC trials was >65 years [4,14]. Older patients may not tolerate standard or investigational cytotoxic chemotherapies as well as younger patients [15]. Selection bias may limit extrapolation of results from non-randomized trials as patients treated in specialized referral centers may be younger and with better performance status than the general patient population. Therefore, we restricted our investigation to elderly patients (defined as age ≥ 65 years) to increase the feasibility and relevance of our results to the management of AIPC.

## Methods

### Study design

This study was a multi-center, non-randomized phase II study. The primary endpoint was best overall response and response duration for elderly patients with AIPC. Secondary endpoints were changes in PSA levels, safety, and changes in patient reported health-related quality of life measures. At entry, patients were categorized according to extent and type of metastatic disease (bone, visceral/lymph nodes, or both) for later response assessment.

### Eligibility

Patients were required to have histologically confirmed adenocarcinoma of the prostate progressed following primary or secondary hormonal manipulations. Disease progression was defined as at least two successive increases in serum PSA (> 20% rise from baseline value) taken at least two weeks apart or documented soft tissue or osseous progression demonstrable on imaging studies. Prior hormonal treatment (flutamide, ketoconazole, diethylstilbesterol) must have been discontinued at least four weeks prior to study treatment (6 weeks for bicalutamide). Other entry criteria included: age ≥ 65 years; Karnofsky Performance Status (KPS) ≥ 70%; castrate levels of testosterone (≤ 50 ng/ml) maintained with medical or surgical castration; no prior treatment with cytotoxic chemotherapy; peripheral neuropathy ≤ grade 1; and appropriate renal, hepatic, and hematologic function consistent with package insert for docetaxel. The Institutional Review Boards at participating institutions approved this protocol and all patients were counseled regarding the benefits, risks, and alternatives to study participation. All patients signed written informed consent forms before any study related procedures were performed.

### Treatment

Treatment consisted of docetaxel 60 mg/m^2 ^IV over 60 minutes every 21 days and erlotinib 150 mg orally daily for 21 days. Patients received dexamethasone 8 mg orally twice daily for 3 days beginning one day prior to the docetaxel infusion. Anti-emetics were administered as per institutional standard of care.

A complete blood count, PSA, AST, ALT, alkaline phospatase, total bilirubin, BUN, creatinine, and electrolytes were obtained at baseline and prior to each cycle of therapy. Patients were evaluated with cross sectional imaging of chest, abdomen, and pelvis and radionucleotide bone scintigraphy at baseline and after every 3 cycles. An electrocardiogram was obtained at screening.

Patients without objective progression of disease (at least stable bone scans and/or stable disease by RECIST criteria) following 9 cycles of docetaxel/erlotinib were allowed to continue on 3 weeks cycles of erlotinib therapy administered alone for up to 8 additional cycles.

### Dose modifications

For erlotinib, dose reductions to 100 mg, 50 mg, or 25 mg, were required for moderate to severe (≥ grade 3) keratitis, rash, or diarrhea. Dose interruptions of erlotinib for up to two weeks were allowed as clinically indicated for erlotinib-related toxicities. For docetaxel, a 25% dose reduction for hematologic toxicities (neutropenia and thrombocytopenia ≥ grade 3) was allowed. Alternatively, myeloid growth factor support for secondary prophylaxis of neutropenia was permitted without dose modification. Dose interruption followed by a subsequent 25% docetaxel dose reduction was required for liver enzyme abnormalities including total bilirubin > upper limit of normal (ULN), alkaline phosphatase > 5 × ULN, and alanine transferase >5 × ULN. Dose reductions were also required for neuropathy ≥ grade 2 and other toxicities ≥ grade 3. Dose delays were required if stomatitis was present on day 1. If moderate to severe (≥ grade 3) stomatitis occurred during any cycle, then subsequent docetaxel doses were reduced by 25%.

### Toxicity and response criteria

The National Cancer Institute Common Toxicity Criteria (version 2.0) was used to evaluate patient toxicity during each cycle. Adverse events were reported to authorities as required by institutional and regulatory guidelines. Every effort was made to administer the regimen at full dose, as tolerated. Standard supportive care measures were employed according to routine local practice.

At entry, patients were classified by disease site according to the presence of measurable lesions by RECIST criteria or non-measurable lesions (by bone scan only) [16]. Patients were evaluated after 3 cycles with bone scans and/or cross sectional imaging as clinically indicated. For patients with measurable disease, response was assessed by standardized RECIST criteria [16]. For patients with bone-only disease, response was assessed by a combination of an improvement in non-measurable lesions (on bone scan) and a reduction in PSA levels (≥ 50%) sustained for at least 21 days. Patients with at least stable disease were continued on therapy. Additional response evaluations were performed after cycles 6, 9, 13, and at study completion (following cycle 17 or at early termination).

Serial measurements of PSA were obtained at baseline and before every cycle. Criteria for response for declines in PSA were based on the guidelines from the PSA working group [17].

Patient reported quality of life outcomes were measured with the FACT-P questionnaire at baseline (day 1 prior to study treatment) and before every treatment cycle. Patients were also asked to complete the FACT-P at the early termination visit, if applicable.

### Statistical considerations

The primary outcome variable was response for each patient as classified by measurable or non-measurable disease. Secondary end points included toxicity, patient reported quality of life, and survival. The study was powered to both estimate the true percent of responders with a tolerance of +/- 23%, based on an exact 95% confidence interval, and to test the null hypothesis of a 5% response versus >5% response with 90% power with a significance level of 0.05. Confidence intervals for the proportion responders were computed using exact binominal confidence intervals. Changes in mean FACT-P scores from baseline to cycle 3 and baseline to end-of-study visit were computed with two-sided Student's t-tests. The Kaplan-Meier curve and mean survival were calculated with S-Plus (version 5; Insightful).

## Results

### Patient characteristics

Between November 2002 and July 2005, 22 patients entered the study at three sites. Patient baseline characteristics are summarized in Table 1. Patient characteristics included a median age of 73.5 years (range 65–80 years) and median performance status of 90 (range 70–100). Primary therapy included radical prostatectomy alone in 6 patients, radiotherapy alone in 2 patients, primary androgen-deprivation therapy (ADT) in 7 patients, and combined prostatectomy and local radiotherapy in 7 patients. The median duration of ADT was 60 months (range 8–132 months) and most patients progressed after a median of 2 secondary hormonal agents (range 0–5). The median pre-treatment PSA was 218 ng/ml. Fourteen had metastatic disease in bone only, 2 patients had soft tissue disease only, and 6 patients had both bone and soft tissue disease.

**Table 1:** Baseline patient characteristics

	**Mean (SD)**	**Median (range)**
Age (years)	74 (5.1)	74 (65–80)
KPS	87.7 (9.7)	90 (70–100)
Hemoglobin (g/dl)	12.1 (1.3)	12.1 (10.0–14.3)
PSA (ng/ml)	665 (1230)	218.3 (9–5754)
Alkaline phosphatase (U/l)	223 (208)	126.5 (51–768)
Duration of androgen deprivation (months)	65.8 (36.3)	60 (8–132)
Secondary hormonal manipulations	2.4 (1.5)	2 (0–5)
		
Primary Therapy	**Number**	
Prostatectomy	13	
Prostatectomy and radiation	7	
Radiation alone	2	
Androgen-deprivation therapy alone	7	
		
Sites of Disease		
Bone only	14	
Visceral only	2	
Both	6	

### Study treatment delivered

A total of 121 cycles of docetaxel with erlotinib were administered to 22 patients. The median number of treatment cycles delivered per patient was 6 (range 1–17). During combination treatment, there were 10 dose reductions and 12 dose delays of docetaxel and 3 dose reduction and 3 dose delays of ≥ 7 days of erlotinib due to adverse events. The average dose intensity of docetaxel was 17.8 mg/m^2^/week (standard deviation [SD], 3.6). The treatment plan specified erlotinib monotherapy after completion of 9 cycles of docetaxel/erlotinib combination treatment with stable disease or better response. Six patients remained on study after cycle 9 to receive a total of 23 cycles of erlotinib monotherapy. There were no dose reductions or delays for erlotinib monotherapy.

Reasons for withdrawal from the study included disease progression in 8 patients, toxicity in 6 patients, and investigator discretion in 6 patients. Two patients completed study drug treatment (after 17 cycles) with stable disease.

### Response and overall survival

All patients entered in the trial were evaluable by PSA criteria (Table 2). Of the 22 patients enrolled, 5 had ≥ 50% decline in PSA (23%, 95% CI 8–45%). None of 8 patients with measurable disease had objective response by RECIST criteria and 1 of 20 patients had a clinical response by composite of bone and PSA criteria (5%, 95% CI 0.1–25%).

**Table 2:** Best overall response by criteria.

Criteria	Response/Evaluable	% (95% CI)
PSA	5/22	23 (8–45)
Measurable	0/8	0 (0–31)
Bone disease	1/20	5 (0–25)

**Figure 1:**
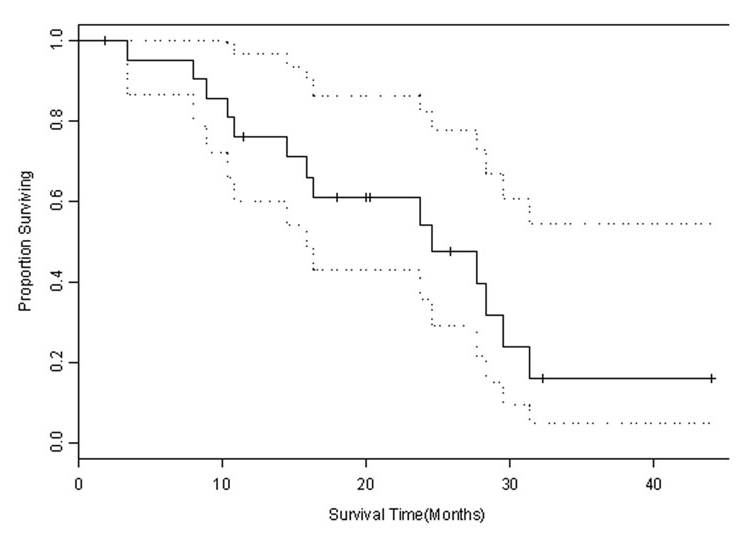
**Estimate of overall survival**. Kaplan-Meier graph showing proportion surviving over time (solid line) with estimates of 95% confidence (dotted line).

With a median duration of follow-up of 19.0 months, a median overall survival of 24.6 months was observed (Figure 1). Five patients remain alive. There were no deaths during the active treatment phase of the trial, but 14 patients died in follow-up. Three patients were lost to follow-up.

### Toxicity

Common treatment-related toxicities, expressed on a per patient basis, are summarized in Table 3. Anemia was the most common hematologic toxicity observed as mild (grade 1 or 2) in 8 patients and moderate (grade 3 or 4) in 1 patient. Neutropenia was observed as mild (grade 1 or 2) in 3 patients and moderate (grade 3 or 4) 9 patients. There were two episodes of neutropenic fever. The most common non-hematologic toxicities were rash (usually grade 1) in 21 patients and fatigue which was mild (grade 1 or 2) in 17 patients and moderate (grade 3 or 4) in 7 patients. Gastrointenstinal toxicities were also common including taste alteration, nausea, mucositis, and diarrhea (all generally grade 1 or 2). Significant (grade 3 or 4) non-hematologic toxicities (other than fatigue) were rare and included anorexia (3 patients) and diarrhea (2 patients). Other common, mild (grade 1 or 2) toxicities included mucositis, rash, elevated transaminases (AST or ALT), dizziness, hypertension, and ocular irritation.

**Table 3:** Treatment Related Toxicity

	**Grade 1/2**		**Grade 3/4**	
**Toxicity**	**No.**	**%**	**No.**	**%**
**Hematologic**				
Neutropenia	3	14	9	41
Anemia	8	36	1	5
Febrile neutropenia	0	0	2	9
Leukopenia	3	14	4	18
Lymphopenia	4	18	4	18
				
**Non-hematologic**				
Fatigue	21	95	1	5
Anorexia	17	77	7	32
Diarrhea	16	72	2	9
Mucositis	14	64	0	0
Rash	13	59	0	0
Liver enzyme elevation (AST)	12	55	0	0
Liver enzyme elevation (ALT)	9	41	0	0
Dizziness	9	41	0	0
Hypertension	7	32	0	0
Ocular irritation	7	32	0	0

### Patient reported quality of life measures

Patients were asked to complete the FACT-P questionnaire before treatment at cycle 1 (baseline), cycle 3, and at the end-of-study visit (occurring variably before cycles 3 through 17). The mean ± SD FACT-P scores observed at baseline, cycle 3, and end-of-study were112 ± 11 (22 patients); 112 ± 14 (18 patients), 106 ± 17 (20 patients), respectively. No significant differences were observed in pair-wise comparisons between mean FACT-P at baseline and cycle 3 and baseline and end-of-study (p > 0.05).

## Discussion

The development of specific inhibitors of the HER-kinase axis represents an important advance in oncology. In many pre-clinical models, inhibitors of HER-kinase pathway are found to be additive or synergistic when combined with cytotoxic agents. This study demonstrates that docetaxel (60 mg/m^2 ^every 21 days) with erlotinib 150 mg daily is active and well-tolerated therapy for elderly patients with AIPC.

This trial was designed and executed before the results of randomized trials of erlotinib or geftinib (a related EGFR-tyrosine kinase inhibitor) with cytotoxic chemotherapy for other solid tumors became available. In non-small cell lung cancer, four large randomized trials of erlotinib or geftinib with first-line cytotoxic chemotherapy demonstrated no additive anticancer effect of the combination [18-21]. Subsequently, erlotinib was shown to increase overall survival over supportive care for patients previously treated with cytotoxic chemotherapy [12]. In pancreatic cancer, a recent trial showed a small, but statistically significant, improvement in overall survival with erlotinib added to gemcitabine versus gemcitabine alone in the first-line setting [11]. These data suggest that, in some settings, EGFR-tyrosine kinase inhibitors may produce a modest increase in anti-cancer efficacy when added to cytotoxic chemotherapy. However, additional data are needed to better understand patient selection and sequencing for these agents.

The toxicity of docetaxel with erlotinib needs to be considered in the context of published data for docetaxel and erlotinib used as monotherapy in comparable patient populations. Rash and diarrhea are the main dose-limiting toxicities of erlotinib and both were commonly observe in this trial, though generally mild [22]. Otherwise, the non-hematologic toxicities of docetaxel with erlotinib appear comparable to docetaxel montherapy as reported in the TAX-327 trial [4]. However, the frequency of dose-modifications and patient withdrawals in this study raises concerns. In the present study, the median number of cycles delivered per patient was 6 (of 9 cycles intended) compared to 9.5 (of 10 cycles intended) in TAX 327 [4]. Further, it is notable that 4 patients withdrew before the first scheduled evaluation (at cycle 3) due to toxicity or patient/physician preference. These dose reductions and delays occurred despite a lower intended dose intensity of docetaxel at 60 mg/m^2 ^every 21 days compared to 75 mg/m^2 ^every 21 days in TAX-327. These data may raise concerns regarding the ability to deliver sufficient doses of docetaxel to affect the overall survival advantage associated with docetaxel-based therapy for AIPC.

The key results of this trial should also be considered in the context of the docetaxel monotherapy arm in TAX-327 [4]. We observed a PSA decline of ≥ 50% in 23% (95% CI 8–45%) which just overlaps with the PSA decline rate of 45% (95% CI 40–51%) observed in TAX-327. The absence of objective responses in 8 patients with measurable disease (0%, 95% CI: 0–31%) in our trial is not surprising considering an objective response rate of 12% (95% CI 7–19%) in TAX-327. Perhaps most significantly, the median overall survival of 24.6 months compares favorably with a median survival of 18.9 months observed in TAX-327 despite a generally older patient population (mean age 74 years in this report versus 68 years in TAX-327) and decreased intended dose intensity of docetaxel (20 mg/m^2^/week in this report versus 25 mg/m^2^/week in TAX-327).

## Conclusion

In summary, we found docetaxel with erlotinib to be a relatively well-tolerated regimen in this elderly patient population. Our data suggests that the overall survival of patients treated with docetaxel and erlotinib appears comparable docetaxel monotherapy despite an apparent increase in toxicity. A randomized study would be needed to determine if the toxicity of the docetaxel and erlotinib results in a demonstrable benefit in terms of overall survival.

## Competing interests

MEG served on an advisory board for OSI Pharmaceutics Inc. (<$10,000). Other authors have no competing interests to report.

## Authors' contributions

DBA designed the study and was primary investigatory of the clinical protocol. MEG analyzed the data, drafted and finalized the manuscript, and coordinated its submission. MEG, CT, AP, and DBA enrolled patients in the clinical protocol. All authors read and approved the final manuscript.

## Pre-publication history

The pre-publication history for this paper can be accessed here:


